# Characterization of peripheral white blood cells transcriptome to unravel the regulatory signatures of bovine subclinical mastitis resistance

**DOI:** 10.3389/fgene.2022.949850

**Published:** 2022-09-20

**Authors:** Jinyan Yang, Yongjie Tang, Xueqin Liu, Jinning Zhang, Muhammad Zahoor Khan, Siyuan Mi, Chuduan Wang, Ying Yu

**Affiliations:** ^1^ Laboratory of Animal Genetics and Breeding, Ministry of Agriculture and Rural Affairs of China, National Engineering Laboratory of Animal Breeding, College of Animal Science and Technology, China Agricultural University, Beijing, China; ^2^ Department of Animal Sciences, Faculty of Veterinary and Animal Sciences, University of Agriculture, Dera Ismail Khan, Pakistan

**Keywords:** bovine subclinical mastitis, lncRNA, mRNA, alternative splicing, single nucleotide polymorphism, peripheral blood transcriptome

## Abstract

Subclinical bovine mastitis is a pathogenic infection of the breast characterized by a marked decrease in milk production and quality. As it has no obvious clinical symptoms, diagnosis and treatment are challenging. Therefore, searching for biomarkers in cows’ peripheral white blood cells is valuable for preventing and treating subclinical mastitis. Thus, in this study, the transcriptome of peripheral blood from healthy and subclinical mastitis cows was characterized to find the regulatory signatures of bovine subclinical mastitis using RNA-seq. A total of 287 differentially expressed genes (DEGs) and 70 differentially expressed lncRNAs (DELs) were detected, and 37 DELs were documented near known Quantitative Trait Loci (QTL) associated with the mastitis of cows. Bioinformatic analysis indicated that lncRNAs MSTRG25101.2, MSTRG.56327.1, and MSTRG.18968.1, which are adjacent to the SCS QTL and SCC QTL, may be candidate lncRNAs that influence the pathogenesis of mastitis in cows by up-regulating the expression of genes *TLR4*, *NOD2*, *CXCL8*, and *OAS2*. Moreover, the alternative splicing (AS) pattern of transcriptional sequence differences between healthy cows and subclinical mastitis cows suggested a molecular mechanism of mastitis resistance and susceptibility. A total of 2,212 differential alternative splicing (DAS) events, corresponding to 1,621 unique DAS genes, were identified in both groups and significantly enriched in immune and inflammatory pathways. Of these, 29 DAS genes were subject to regulation by 32 alternative splicing SNPs, showing diverse and specific splicing patterns and events. It is hypothesized that the *PIK3C2B* and *PPRPF8* splice variants associated with AS SNPs (rs42705933 and rs133847062) may be risk factors for susceptibility to bovine subclinical mastitis. Altogether, these key blood markers associated with resistance to subclinical mastitis and SNPs associated with alternative splicing of genes provide the basis for genetic breeding for resistance to subclinical mastitis in cows.

## 1 Introduction

Bovine mastitis is the foremost production and major economic burden confronted by the global dairy industry (Hoque et al., 2019). The disease may be asymptomatic or symptomatic (subclinical or clinical mastitis), persistent (chronic), or nonpersistent. Infectious agents such as *Staphylococcus aureus* (*S. aureus*), *Streptococcus agalactiae*, and *Mycoplasma spp* as well as environmental pathogens such as *Escherichia coli*, *Klebsiella pneumoniae,* and *Enterobacter aerogenes* are the main causes of mastitis in cattle (Gao et al., 2017; Hoque et al., 2020, 2019). In countries such as China, 10–40% of mastitis cases are caused by *S. aureus* (Wang et al., 2018). These bacteria enter the mammary gland and are recognized by the interaction of their pathogen-associated molecular patterns (PAMP), and the resulting induced inflammatory response leads to the shedding of somatic cells (SC) into the milk. It has been demonstrated that both the number of somatic cells count (SCC) and changes in their gene expression are associated with physiological processes in the mammary gland and bacterial infection (Leitner et al., 2000), with 200,000 cells/mL being used as the best cut-off point to distinguish between infected and uninfected (Schepers et al., 1997; Goncalves et al., 2018).

Blood is a mixture of various immune cells, such as lymphocytes, neutrophils, and monocytes, which have an advantage in reflecting traits such as immunity and disease resistance in cows (Chaussabel, 2015). Also, circulating leukocytes in the blood play a key role in the onset, development, and regression of mastitis, as they are the primary source of immune cells attracted to the mammary gland during infection (Cheng et al., 2021). Most importantly, blood is an easily accessible and least damaging tissue sample compared to bovine mammary epithelial cells, which can accurately reflect the physiological status and health of cows during lactation (Bai et al., 2016). Thus, gene expression profiles from blood offer new opportunities to clarify the mechanisms underlying the complex traits of cows. Blood biomarkers play an important role in characterizing the disease state of animals, and many studies have reported altered mRNA and long non-coding RNA abundance in the mammary gland after mastitis, with some stable markers such as lncRNA XIST (Ma et al., 2019a), lncRNA-TUB (Wang H. et al, 2019), LRRC75A-AS1 (Wang et al., 2020b) and lncRNAs PRANCR (Mi et al., 2021) were identified, but the role of gene transcription and complex networks in the blood remains unclear.

The diversity of functional protein products is mostly attributed to gene alternative splicing. Alternative splicing is a fundamental mechanism by which introns in pre-mRNAs are clipped and exons are bonded together in different configurations, leading to alterations in the structure of major transcripts (Baralle and Giudice, 2017). The previous findings showed that well over 60% of human genes undergo substitutive splicing (Modrek et al., 2001) which has a strong association with diseases (Faustino and Cooper, 2003; Urbanski et al., 2018; Yang et al., 2019). In cows, one study found that 4,567 of 21,755 bovine genes are alternatively spliced, and the most common AS event is exon skipping (Chacko and Ranganathan, 2009). These AS events are strongly associated with disease resistance in cows. For example, two differential alternative splicing (DAS) events in the genes *EXOC7* and *KIF2C* affect protein functional domains and are associated with the susceptibility to *Mycobacterium avium* subspecies paratuberculosis (Li et al., 2022). Furthermore, the specific alternative splicing patterns exhibited in genes *SLAMF7* (Ju et al., 2012a), *BOLA-DQA2* (Hou et al., 2012), and *CD46* (Wang et al., 2016) in mammary gland tissues of cows were associated with mastitis resistance in cows.

Moreover, 15% of point mutations on genomic DNA that lead to genetic diseases impact pre-mRNA splicing (Wang et al., 2016). Several splicing-related single nucleotide polymorphisms (SNP) may directly alter the coding region, leading to aberrant alternative splicing that affects the disease phenotype (Wang et al., 2014; Ren et al., 2021). In cattle, a study found that an independent spontaneous splice site variant in *COL2A1* (g.32473300 G > A) was most likely responsible for chondrodysplasia during early fetal development in cows. Similarly, the SNP g.5880C > T, SNP g.18174A > G, and SNP g.10766T > C might affect the binding with splicing-related factors, subsequently causing the production of aberrant splice variant *HMGB3-TV1* (Li et al., 2013; Wang et al., 2014)*, NCF4-TV* (Ju et al., 2015), and *NCF1-TV1* (Zhang et al., 2015), thus increasing somatic cell scores in cows.

In the current study, we used RNA-seq to characterize gene expression patterns in the peripheral white blood cells transcriptome of healthy cows and subclinical mastitis cows. Furthermore, we obtained several candidate blood-based transcriptional biomarkers based on the comprehensive analysis of gene function annotation and gene expression patterns. Afterward, we further analyzed alternative splicing events at the transcription level and searched for splicing-associated mutations to elucidate the molecular regulatory mechanisms of bovine subclinical mastitis resistance.

## 2 Materials and methods

### 2.1 Sample collection and preparation

Three healthy cows and three subclinical mastitis cows were chosen according to three consecutive months of SCC records and other related phenotypes. In this study, cows with milk SCC values below 100,000 cells/mL were regarded as healthy cows. In contrast, cows with SCC values in the range of 200,000 cells/mL and 500,000 cells/mL were considered to be suffering from subclinical mastitis cows. For similarity in the biological background, the cows in this study had similar lactation days and all calved one litter (Supplementary Table S1).

About 20 mL of anticoagulant blood sample was collected from each cow. About 10 mL of anticoagulant blood sample was centrifuged at 3,500 r/min for 15 min and the middle leukocyte layer was collected and stored in 1 mL of TRIzol at -80 °C for RNA extraction. In addition, about 2 mL blood sample was sent to Jinhaikeyu company for HP testing (Beijing, China). A further amount of blood was used for genotyping at GGP Bull 150K BeadChip (Neogene, Lansing, MI, United States).

### 2.2 Hematological parameters

A Sysmex K-4500 automated hematology analyzer (Sysmex Corporation, Kobe, Japan) was used to test 24 hematological parameters, including White Blood Cells (WBC), Red Blood Cells (RBC), Hemoglobin (HGB), Red blood cell-specific volume (HCT), Mean Corpuscular Volume (MCV), Mean Corpuscular Hemoglobin (MCH), Mean Corpuscular Hemoglobin Concentration (MCHC), Platelet count (PLT), Neutrophil Ratio (NETU%), Neutrophil count (NETU#), Lymphocyte Ratio (LYMPH%), Lymphocyte count (LYMPH#), Monocyte Ratio (MONO%). Monocyte count (MONO #), Eosinophil Ratio (EO%), Eosinophil count (EO#), Basophil Ratio (BASO%), Basophil count (BASO#), Platelet Distribution Width (PDW), Mean Platelet Volume (MPV), Red cell distribution width - stand error (RDW-SD), Red blood cell distribution width - coefficient of variation (RDW- CV), Platelet-Large Cell Ratio (P-LCR), Platelet cubic metric distribution width (PCT).

### 2.3 RNA extraction and RNA sequencing

Total RNA of peripheral white blood cells for each cow was extracted with the TRIzol reagent (Invitrogen, Carlsbad, CA, United States) following the manufacturer’s procedures. The concentration of RNA was estimated using the NanoDrop 2000 (ThermoFisher Scientific, Waltham, MA, United States) and the RNA Nano 6000 Assay Kit of the Bioanalyzer 2,100 system was used to assess RNA integrity. The RNA quality was checked for contamination and degradation by a 1% agarose gel. Then, qualified RNA was used to construct RNA-seq libraries. Finally, cDNA libraries were sequenced with 150 bp paired-end reads from Illumina NovaSeq 6,000 platform (Novogene, Beijing, China).

### 2.4 RNA-Seq data analysis

#### 2.4.1 Reads quality control and mapping

The quality of the raw data was evaluated with FastQC version 0.11.8. The clean data were obtained by using Trimmomatic version 0.38 to filter out reads with adapter sequences and low-quality reads from the raw reads (Bolger et al., 2014). Specifically, remove splice sequences, bases with mass less than three at the 5′ and 3′ ends of reads, and all bases with average mass less than 15 in the window; trim reads with sequence length less than 36. Subsequently, paired-end reads from each sample were aligned to the reference genome using STAR version 2.7.7a (Dobin et al., 2013). The generation of SAM files was sorted into BAM files using SAMtools version 1.9 (Li et al., 2009). Subsequently, each sample was assembled and merged using StringTie version 1.3.5 (Pertea et al., 2015).

#### 2.4.2 Prediction novel lncRNA

In the current study, strict filtering conditions were set on the annotated transcripts to obtain novel lncRNA transcripts. Firstly, transcripts shorter than 200bp and less than two exons were discarded. Then, transcripts with the class codes “i" (intronic lncRNA, ilncRNA), “u" (Intervening lncRNAs, lincRNA), and “x" (antisense lncRNA, lncNAT) were preserved. Finally, protein-coding potential predictions were performed using the Coding-Non-Coding Index (CNCI) (Sun et al., 2013), ORF Length and GC content (LGC) (Wang G. et al., 2019), Coding Potential Calculator (CPC) (Kang et al., 2017), and Predictor of Long non-coding RNAs and messenger RNAs based on an improved K-mer scheme (PLEK) (Li et al., 2014), and only those transcripts at the intersection of the four software tools 132 were selected as novel lncRNA transcripts.

#### 2.4.3 Quantification and Identification of Differentially Expressed Genes

Transcripts were quantified using HTSeq-counts version 2.0.1 and results were expressed as read counts (Anders et al., 2015). Subsequently, normalization of reads and differential expression analysis of reads were performed using the DESeq2 (Love et al., 2014). Differentially expressed genes (DEGs) and differentially expressed lncRNAs (DELs) were screened with the criteria of *p*-value < 0.05 and log2 (fold change) > 1.5.

#### 2.4.4 Prediction of the *cis* and *trans*-target genes of lncRNAs

To explore the functions of lncRNAs, we simultaneously predicted the *cis*- and *trans*-target genes of lncRNAs. Protein-coding genes near the transcript positions of lncRNAs (100k upstream and downstream) were selected as *cis*-target genes of lncRNAs with the BEDTools version2.1.2 (Quinlan and Hall, 2010). *Trans*-target genes were predicted based on Pearson correlation coefficients between DELs and DEGs calculated by the R package-Hmisc, with screening criteria of *r* > 0.98 and *p*-value < 0.05.

#### 2.4.5 Gene Alternative Splicing Analysis

In the alternative splicing analysis, alternative splicing events were detected using rMATS version 4.1.1 (Shen et al., 2014). In this study, five alternative splicing patterns, namely exon skipping (ES), retained intron (RI), mutually exclusive exon (MXE), alternative 5′ splice site (A5SS), and alternative 3’ splice site (A3SS), were detected from healthy cows and subclinical mastitis cows using rMATS software version 4.1.1. Statistical verification (FDR <0.05) was performed when the differences in isoform ratio (IncLevelDifference) of different alternative splice genes (DAS genes) exceeded the defined threshold of 0.01%. Static images of sarcograms were generated outside of IGV using sarcograms, which is a python tool that is part of the MISO package.

#### 2.4.6 Functional annotation of SNPs

In this study, all animals were genotyped using the GGP Bull 150K BeadChip (Neogene, Lansing, MI, United States) and the effects of all SNPs were annotated by ensemble Variant Effect Predictor (VEP) to screen for SNPs associated with alternative splicing. Subsequently, SNPs were localized to target genes based on positional information to identify candidate SNPs associated with alternative splice genes.

### 2.5 Functional enrichment analysis

Kyoto Encyclopedia of Genes and Genomes (KEGG) enrichment analysis and gene ontology (GO) analysis of DEGs, target genes of DELs, and DAS genes were performed on the KOBAS 3.0 online website (http://kobas.cbi.pku.edu.cn/kobas3/).

### 2.6 Construction interaction network of DELs, DEGs, and pathways

A regulatory network of DELs, target genes of DELs, and pathways were constructed using Cytoscape version 3.5.1 (Yin et al., 2020)

### 2.7 Statistical analysis

Linear regression analysis was performed using GraphPad Prism (version nine; GraphPad Software, San Diego, CA, United States) and Student’s *t*-test was used to examine significant differences in the expression levels of lncRNAs and mRNAs between healthy and subclinical mastitis cows. The ggpubr was used to analyze correlations between routine blood test parameters and standardized read counts of genes.

## 3 Results

### 3.1 Overview of RNA-seq data

RNA-seq was performed on six cDNA libraries using the Illumina NovaSeq 6,000 platform. The total reads mapped to the bovine genome have been summarized in Table 1. A total of 12.79 million raw reads per library and 12.59 million clean-read pairs were generated from all cDNA libraries. The GC contents of the reads ranged from 50 to 52%. The average alignment rate of clean reads to the bovine genome (version: ARS-UCD1.2) was 95.89%, while 90.91% of clean reads were uniquely mapped reads. Reads that uniquely mapped to the reference genome were used for further analysis (Table 1).

**TABLE 1:** Summary of reads mapped to the bovine genome.

Cows	Raw Reads	Clean Reads	Total Mapped Rate (%)	Uniquely Mapped Rate (%)
HY1	20,989,867	20,691,802	96.15	91.51
HY2	19,257,369	18,970,017	96.32	91.39
HY3	24,010,276	23,632,252	96.70	92.13
SM1	21,807,528	21,481,669	96.03	90.67
SM2	23,137,865	22,746,107	96.17	90.44
SM3	18,669,665	18,418,778	93.96	89.31

### 3.2 Identification and characterization of lncRNAs in subclinical mastitis cows vs. healthy cows

**FIGURE 1:**
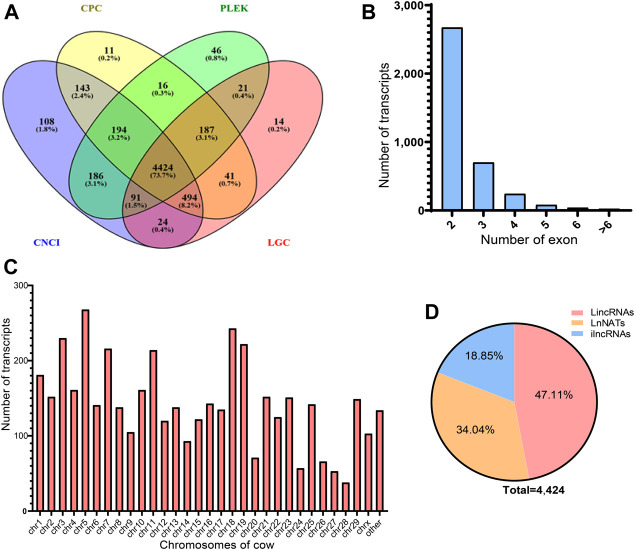
Identification and characterization of lncRNAs in cows with subclinical mastitis and healthy cows. **(A)** The Venn diagram of novel lncRNAs. The intersection part showed the novel lncRNAs predicted by the coding potential prediction software CPC, PLEK, CNCI, and LGC together. **(B)** Exon number of identified lncRNAs transcripts. **(C)** Distribution of identified lncRNAs transcripts on all chromosomes. **(D)** Among 4,421 lncRNA transcripts, the proportion of intronic lncRNA (ilncRNA), Intervening lncRNAs (lincRNA), and antisense lncRNA (lncNAT).

The identification of novel lncRNAs transcripts was followed by a series of criteria. Upon transcript filtering and protein-coding potential prediction, 5,510, 5,664, 5,296, and 5,165 lncRNAs transcripts were predicted by CPC, CNCI, LGC, and PLEK, respectively (Figure 1A). Of particular note, 4,424 lncRNAs transcripts were identified as novel lncRNAs transcripts, which were evaluated by four prediction tools. Of the 4,424 identified lncRNAs transcripts, 76.29% had two or three exons, while 0.52% had more than six exons (Figure 1B).

In the peripheral blood of healthy cows and subclinical mastitis cows, a total of 6,365 lncRNAs were screened, including 1,941 known lncRNAs transcripts. These transcripts were widely distributed in all chromosomes of the bovine genome, and the highest number of transcripts was on chromosome five (Figure 1C). lncRNAs were classified according to their genomic location, with intervening lncRNAs having the highest proportion (47.11%) of all novel lncRNAs, followed by antisense lncRNAs (26.92%) and the lowest percentage of intronic lncRNAs (18.85%) (Figure 1D).

### 3.3 lncRNAs and mRNA expression profile changes in subclinical mastitis cows vs. healthy cows

**FIGURE 2:**
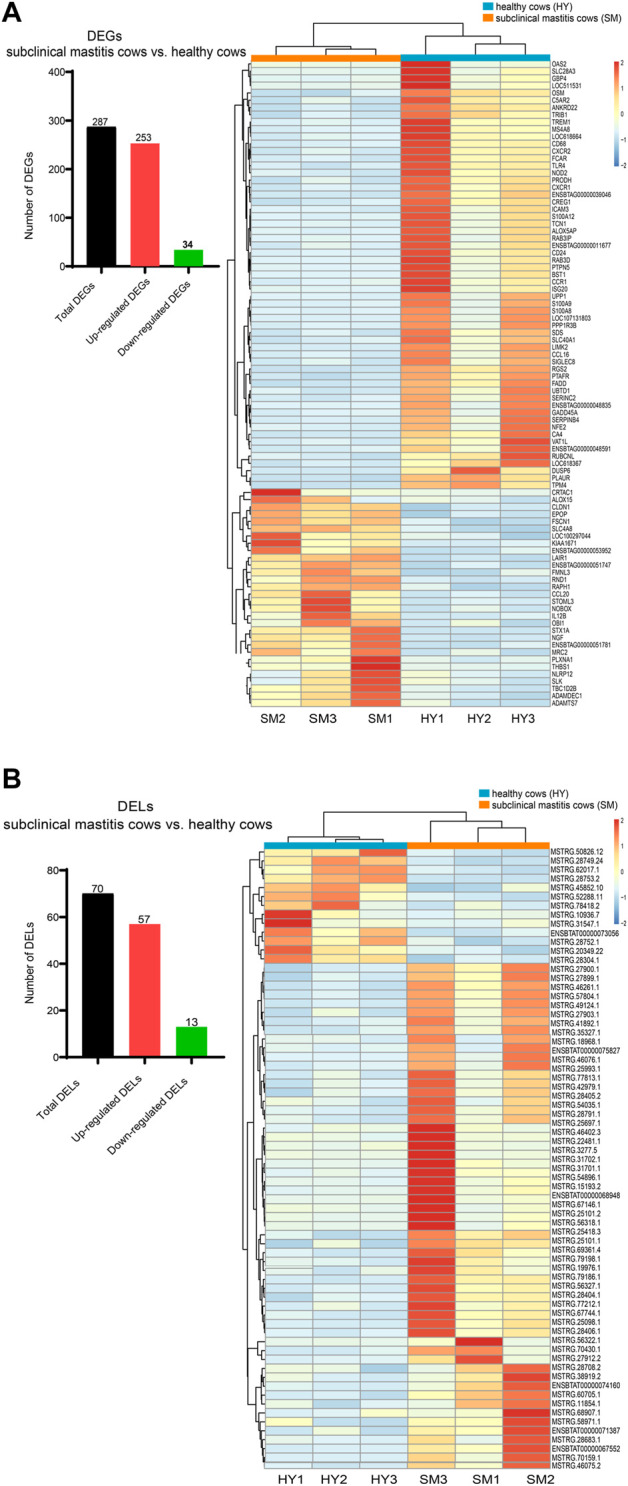
lncRNAs and mRNA expression profile changes in subclinical mastitis cows *vs.* healthy cows. The number **(A)** and the heatmap **(B)** differentially expressed genes (DEGs) and differentially expressed lncRNAs (DELs) in subclinical mastitis (SM) cows *vs.* healthy (HY) cows (*p*-value < 0.05, log2 (fold change) > 1.5).

A total of 287 differentially expressed genes (DEGs) and 70 differentially expressed lncRNAs (DELs) were identified between the two groups (*p*-value < 0.05, log(fold change) > 1.5) (left panel in Figures 2A,B). Compared with healthy cows, 253 genes and 57 lncRNAs were significantly up-regulated, while 34 genes and 13 lncRNAs were significantly down-regulated in subclinical mastitis cows. The clustering results of the heatmaps showed that the expression patterns of lncRNAs and mRNAs were quite different between healthy and subclinical mastitis cows (right panel in Figures 2A,B).

**FIGURE 3:**
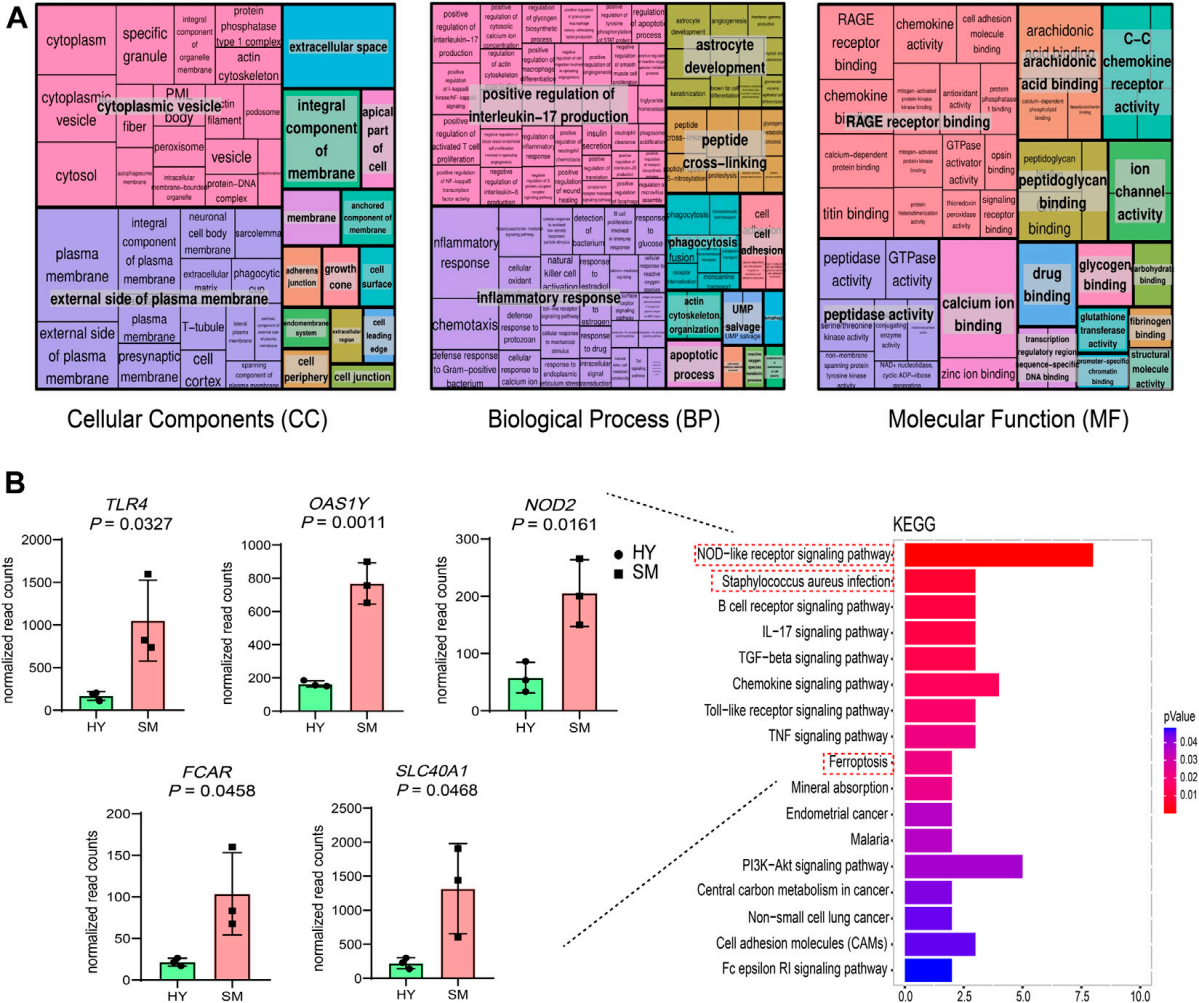
Results of GO and KEGG analysis of 287 differentially expressed genes (DEGs) in subclinical mastitis (SM) cows *vs.* healthy (HY) cows. **(A)** REVIGO summary and visualization of the significant Go terms (*p*-value < 0.05), including 190 GO terms of biological process (BP), 50 GO terms of cellular components (CC), and 56 GO terms of molecular function (MF). **(B)** KEGG pathways of DEGs (*p*-value < 0.05), and expression differences of several DEGs involved in the *S. aureus* infection, cell adhesion molecules, and ferroptosis pathway.

Next, the functions of 287 DEGs were investigated. As shown in Figures 3A, B, 287 DEGs were involved in 301 GO terms (*p*-value < 0.05), which contained 55 molecular compositions (CC), 190 biological processes (BP), and 56 molecular functions (MF). Furthermore, KEGG results of DEGs showed 17 enriched signaling pathways (*p*-value < 0.05) (Figure 3B). Among them, the NOD-like receptor signaling pathway was enriched with the highest number of DEGs (Supplementary Table S2). The genes *FCAR*, *PTAFR*, and *SELPLG* are involved in the *S. aureus* infection pathway, among which *SELPLG* is also involved in the cell adhesion signaling pathway (Supplementary Table S2). Notably, the genes *SLC40A1* and *SLC11A2* were involved in the ferroptosis signaling pathway, and *SLC40A1* was up-regulated (Figure 3B) in subclinical mastitis cows *vs.* healthy cows.

**FIGURE 4:**
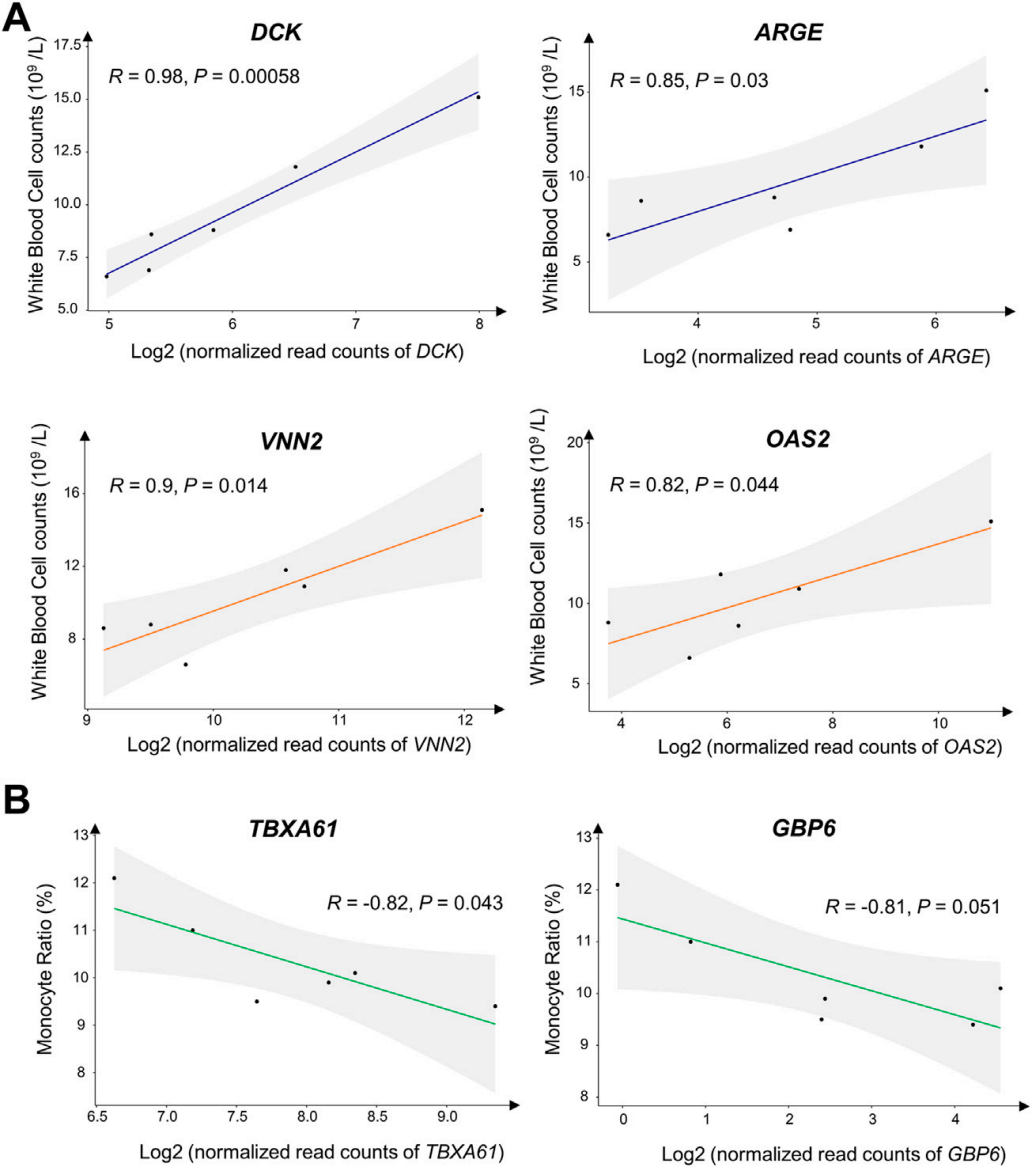
Linear analysis of normalized read counts for several differentially expressed genes (DEGs). and routine blood test parameters. **(A)** DEGs are significantly associated with White Blood Cell (WBC) count (10^9^/L). **(B)** DEGs are significantly associated with Monocyte Ratio (MONO %).

We also investigated the relationship between DEGs and routine blood test parameters and found that the standardized read counts of genes *DCK*, *ARGE*, *VNN2*, and *OAS2* were significantly correlated with White Blood Cell (WBC) count (10^9^/L) (*p*-value < 0.05) (Figure 4A). Similarly, the standardized read counts of genes *GBP6* and *TBXAS1* were significantly correlated with the Monocyte Ratio (MONO %) in peripheral blood (*p*-value < 0.05) (Figure 4B).

### 3.4 Combined DELs and DEGs of healthy cows and subclinical mastitis cows for exploring their co-expression

**FIGURE 5:**
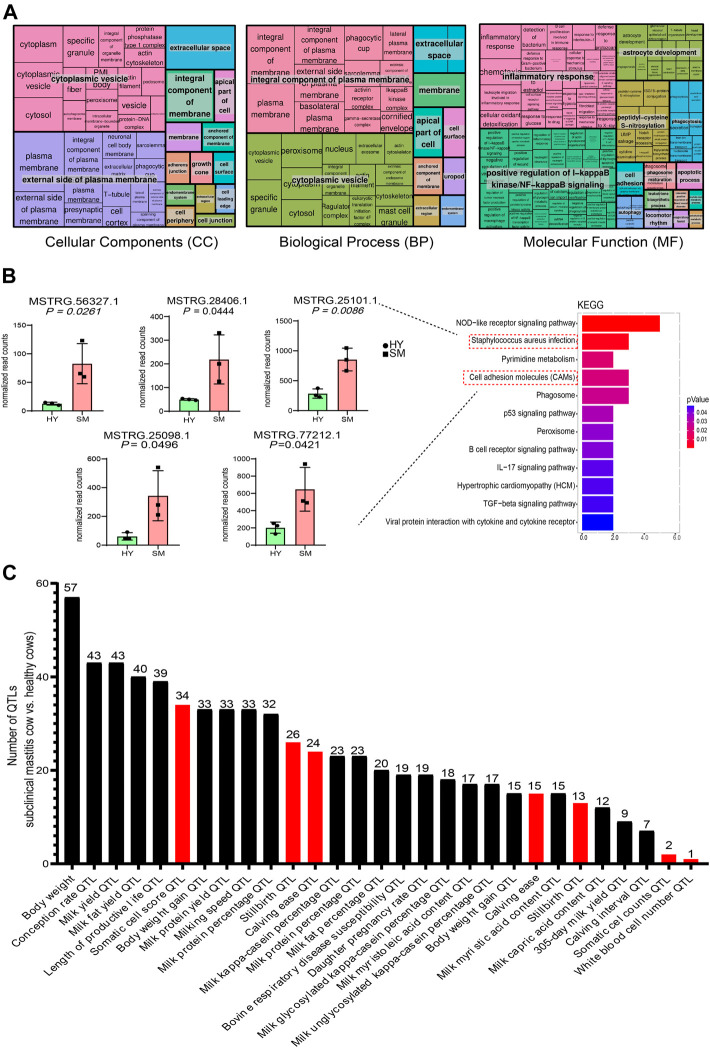
Results of GO and KEGG analysis of lncRNAs *cis*-and *trans*-target genes. **(A)** Significant GO terms (*p*-value < 0.05) for *cis*- and *trans-*target genes of lncRNAs, including 197 GO terms for biological processes (BP), 44 GO terms for cellular components (CC) and 55 GO terms for molecular functions (MF). **(B)** Significant KEGG pathways for *cis* and *trans* target genes of lncRNAs (*p*-value < 0.05), and expression differences of the differentially expressed genes (DEGs) involved in the enrichment of the NOD-like receptor signaling, *S. aureus* infection, and cell adhesion pathways. **(C)** Annotation results of DELs in subclinical mastitis cows compared to healthy cows, with red bars showing DELs associated with reproductive traits and mastitis.

Potential target genes of DELs were predicted by co-localization and co-expression. In this study, 40 *cis*-target genes and 124 *trans*-target genes were predicted (Supplementary Table S2). The results of GO analysis showed that target genes of DELs were involved in 197 BP, 44 CC, and 55 MF (Figure 5A). Most of the target genes were located in cytoplasmic vesicles, outside of the plasma membrane and were involved in the positive regulation of interleukin-17 production, inflammatory response, and cell adhesion (Figure 5A).

The results of the KEGG analysis showed 12 pathways (*p*-value < 0.05) (Figure 5B). Most of these pathways were related to immune responses and inflammation, like NOD-like receptor, p53, IL-17, and *S. aureus* infection pathway. Besides, the genes *FCAR*, *PTAFR*, and *SELPLG* are involved in the *S. aureus* infection pathway, among which *SELPLG* is also involved in the cell adhesion signaling pathway (Supplementary Table S2). Notably, most DELs and their target genes showed the same expression trends (Supplementary Table S2).

### 3.5 Identification of candidate biomarkers associated with subclinical mastitis

In this study, the functions of DELs were further annotated by comparing the genomic positions of DELs and QTL-related regions in Cattle QTLdb. The results showed that 245 DELs were located adjacent to 481 QTLs in Cattle QTLdb (Figure 5C). These QTLs surrounding DELs were associated with milk production performance and reproductive traits, with more than 75% of QTLs being associated with milk (Supplementary Table S3). More importantly, 35 DELs were located adjacent to three QTLs related to mastitis, such as SCC QTL, somatic cell score (SCS) QTL, and white blood cell count QTL (Supplementary Table S3). What’s more, it was found that 32 lncRNAs were adjacent to stillbirth and calving ease QTLs (Supplementary Table S3), of which 15 DELs were also adjacent to white blood cell QTL, SCS QTL, and SCC QTL, such as MSTRG.10101.2, MSTRG.28749.24, MSTRG.46076.1, MSTRG.67501.5, and MSTRG.23805.2, MSTRG.28197.1 (Supplementary Table S3), and so on.

### 3.6 Co-expression networks revealed the regulatory mechanism of lncRNAs in subclinical mastitis

**FIGURE 6:**
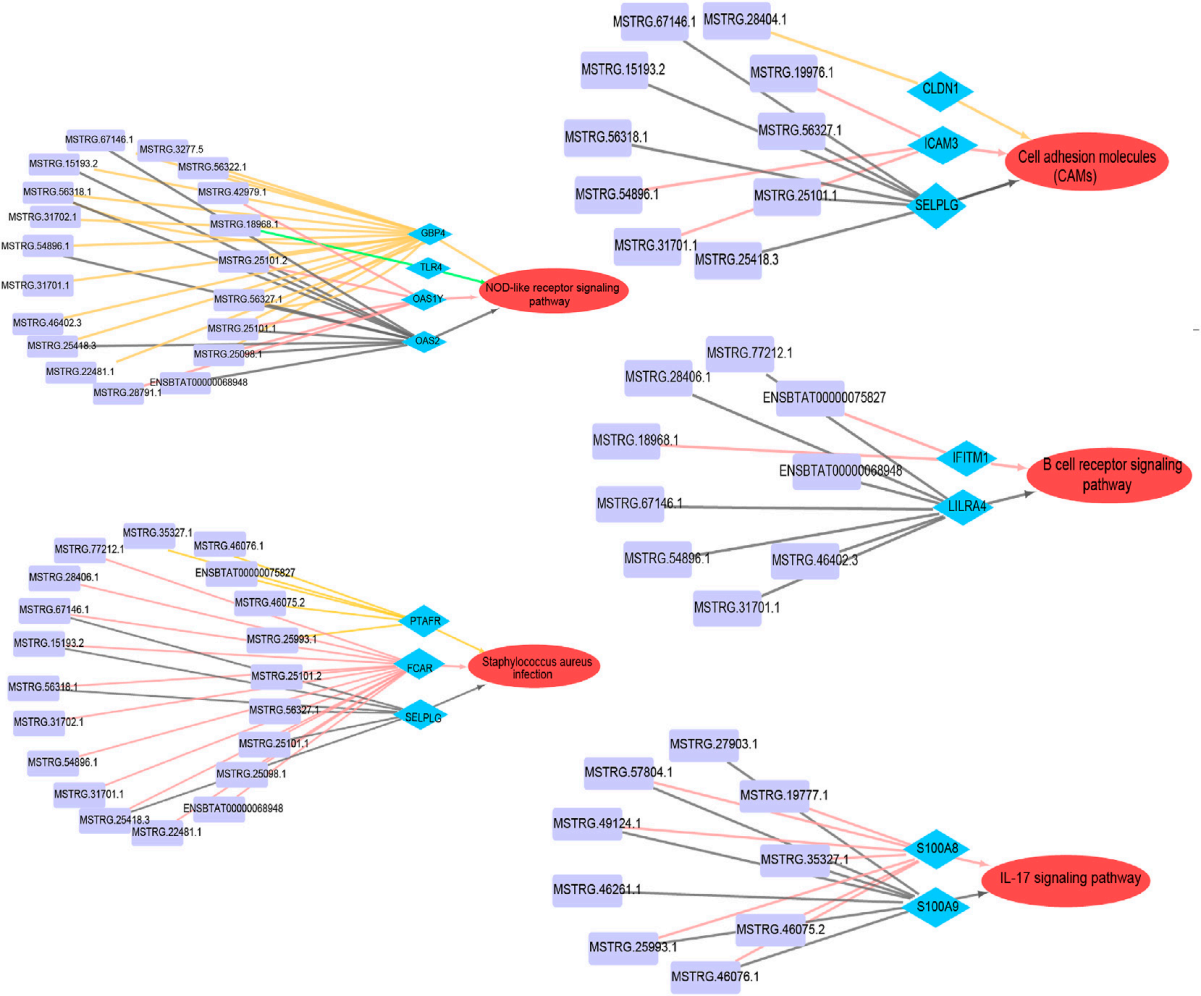
Co-expression networks of lncRNAs *cis*- and *trans*- target gene pathways; In particular, these five pathways were shared by differentially expressed genes (DEGs) and differentially expressed lncRNAs (DELs), including the NOD-like receptor, *S. aureus* infection, and cell adhesion (CAMs), B-cell receptor, and IL-17 signaling pathways. Purple rectangles represents DELs, blue diamonds represents DEGs, red ellipses represents critical paths, and different target relationships in the same regulatory network are distinguished by different colored lines.

Previous studies have shown that lncRNAs may impact disease resistance of livestock and poultry by regulating the expression levels of their *cis*-and *trans*-target genes. In this study, five signaling pathways were shared by DEGs and DELs, including NOD-like receptor, *S. aureus* infection, cell adhesion, B cell receptor, and IL-17 signaling pathways, involving 18 DEGs, 37 DELs, and 7 DEGs. As shown in Figure 6, the network included 117 nodes, 133 edges, and lncRNAs MSTRG.25101.1, MSTRG.25098.1, MSTRG.25418.3, MSTRG.56318.1, MSTRG.15193.2, MSTRG.56327.1, MSTRG.67146.1, and MSTRG.54896.1 may play key nodes to regulate the expression levels of core genes *OAS1Y*, *GBP4*, *OAS2*, *FCAR*, *SELPLG*, *DCK*, and *ICAM3*. This result implied the important role of lncRNAs in bovine subclinical mastitis.

### 3.7 Identification and characterization of total alternative splicing events and differential alternative splicing events

**FIGURE 7:**
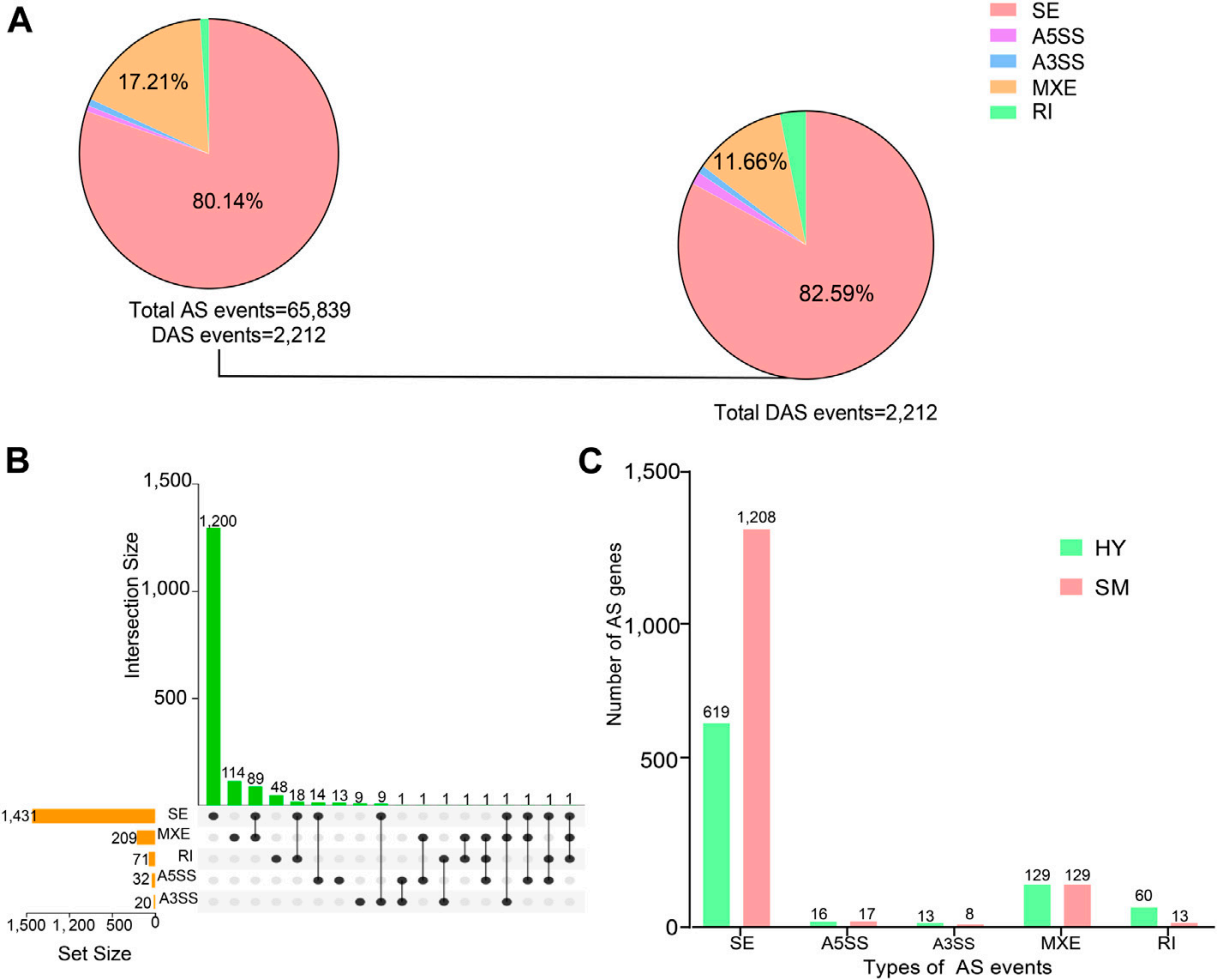
Identification and characterization of total alternative splicing (AS) events and differential AS (DAS) events. **(A)** Distribution of five AS types including skipping exon (SE), alternative 5′ splice site (A5SS), alternative 3′ splice site (A3SS), mutually exclusive exon (MXE), and retained intron (RI) among 65,839 AS events and 2,212 unique DAS events (*FDR* < 0.05). **(B)** Statistical results of shared and specific AS events in two groups. The bar graph on the left shows the number of raw AS events. The connections between points indicated the presence of intersections between corresponding data sets. **(C)** The number of five AS event genes in the healthy and subclinical mastitis cows.

A comprehensive analysis of alternative splicing events in peripheral blood was also performed. As shown in Figure 7A, a total of 65,839 alternative splicing **(**AS) events were found between healthy and subclinical mastitis cows (*FDR* < 0.05), including 2,212 differential alternative splicing (DAS) events. Five patterns of AS consist of exon skipping (ES), retained intron (RI), mutually exclusive exon (MXE), alternative 5′ splice site (A5SS), and alternative 3’ splice site (A3SS). A total of 2,212 events were segregated into 82.59% ES events, 11.66% MXE events, 2.44% A3SS and A5SS events, and 3.30% RI events (Figure 7A). More than two types of AS events were presented in 139 genes, 89 genes showed both MXE and SE events, 18 genes showed both SE and RI events (Figure 7B), and the genes *CCDC51*, *PIK3CG*, *MCEMP1*, *STRADA*, and *ATP2A3* presented three types of AS events.

Further analysis revealed that 60 and 13 AS genes presented RI events, 16 and 17 AS genes presented A5SS events, 13 and 8 AS genes presented A3SS events, and the same number of genes presented MXE events in healthy and subclinical mastitis cows, respectively (Figure 7C). Notably, the number of SE events increased two-fold in subclinical mastitis cows compared to healthy cows (Figure 7C).

### 3.8 Several DAS genes were mastitis-specific and involved in immune, inflammatory response, and reproduction-related pathways

**FIGURE 8:**
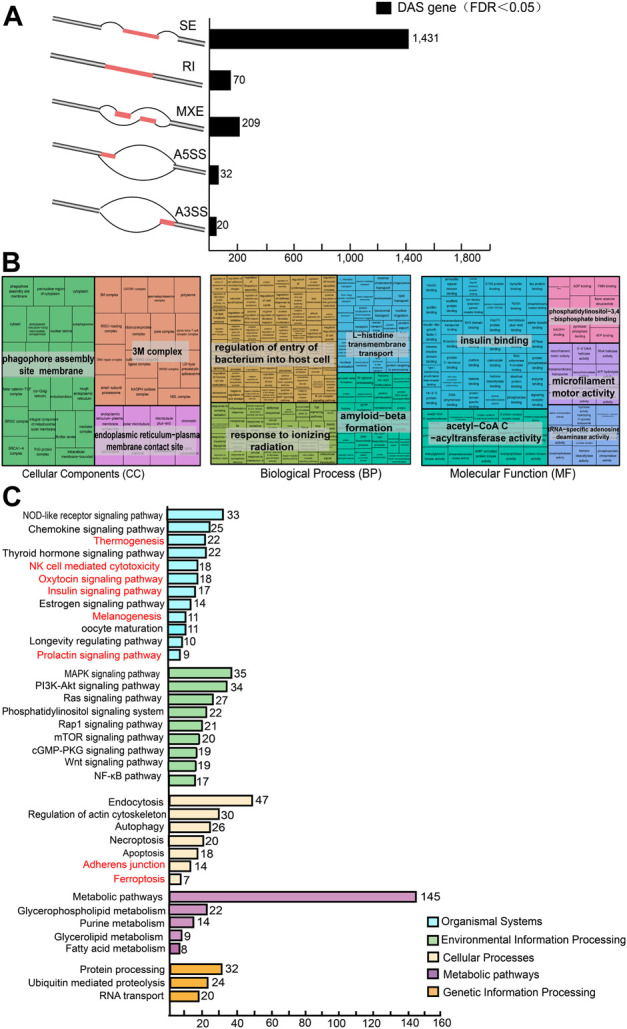
Functional characterization of differential alternative splicing (DAS) genes. **(A)** Distribution of the five essential AS events detected from 1,621 DAS genes, and a schematic illustration of the five AS models, including skipping exon (SE), alternative 5′ splice site (A5SS), alternative 3′ splice site (A3SS), mutually exclusive exon (MXE), and retained intron (RI). **(B)** Significant GO terms for 1,621 DAS genes (*p*-value < 0.05), including 455 GO terms for biological processes (BP), 142 GO terms for cellular components (CC) and 172 GO terms for molecular functions (MF). **(C)** Significant KEGG pathways of 1,621 DAS genes (*p*-value < 0.05), divided into five types, with different colors representing different types.

Through the integration of DAS events that belong to the same gene, 2,212 DAS events were found to correspond to 1,621 unique genes (Figure 8A). Gene Ontology and KEGG pathway analysis were used to characterize the primary functions of AS genes. In all three functional categories, AS genes were annotated to different GO terms, including 142 GO terms for CC, 455 GO terms for BP, and 172 GO terms for MF (Figure 8B), affecting insulin binding, regulation of bacterial entry into host cells, and response to ionizing radiation. KEGG pathway analysis further revealed that DAS genes were enriched in 154 pathways, which were classified into five categories in the KEGG database.

At one hierarchical level of the KEGG results (Figure 8C), a total of 42 signaling pathways associated with the organism system were enriched (Supplementary Table S4). In particular, the highest proportion of signaling pathways related to the immune system were reported, such as chemokines, Toll-like receptor, NOD-like receptor, hematopoietic cell lineage, natural killer cell-mediated cytotoxicity, IL-17, and leukocyte transendothelial migration signaling pathways (Figure 8C), which comprised 134 unique genes, representing 62.62% of the catalog (Supplementary Table S4). As a matter of note, 32 DAS genes were found involved in reproductive trait-related signaling pathways, such as thermogenesis, oxytocin, estrogen, and progesterone-mediated oocyte maturation signaling pathways (Supplementary Table S4). Hierarchical level two showed that 18 signaling pathways are involved in the regulation of mastitis, including MAPK, Ras, cGMP-PKG, NF-κb, phosphatidylinositol, and mTOR, wnt, and TNF pathways (Figure 8C). At the third level, a total of 19 pathways were identified as being related to cellular processes.

Worthy of note, the genes *SLC40A1*, *ACSL6*, *GCLC*, *ACSL5*, *CYBB*, *VDAC3*, and *FTH1* were also enriched in the ferroptosis signaling pathway (Figure 8C). Of these, *SLC40A1* was up-regulated and also enriched in the ferroptosis signaling pathway as DEGs (Figure 3B).

### 3.9 SNPs rs42705933 and rs133847062 may impact mastitis resistance by altering the alternative splicing patterns of *PIK3C2B* and *PRPF8*

From the results of the annotation of ensemble Variant Effect Predictor (VEP) on all SNPs, 51.97% of the SNPs were intronic variants, 34.00% were intergenic variants, and only 0.02% of the variants were associated with alternative splicing (Supplementary Figure S1), which included spliced donor region variants and spliced polypyrimidine tract variants. Ultimately, a total of 32 SNPs associated with alternative splicing were screened, corresponding to 29 unique alternative splicing genes (Table 2), including 23 genes that exhibited SE events, three genes that exhibited MXE events, and three genes that exhibited A5SS events, A3SS events, and RI events (Table 2).

**TABLE 2:** AS SNPs of all SNPs and the AS events of their target genes.

SNP ID	Gene symbol	Location	Allele	*p*-Value	*FDR*	AS Events
rs137001652	*MED15*	17:72,433,357	G	1.35E-10	1.70E-07	MXE
rs134643460	*CDC7*	3:51,880,683	C	9.51E-08	8.14E-06	RI
rs109129224	*FGR*	2:125,704,116	C	9.31E-08	5.58E-05	SE
rs111019940	*NDC1*	3:92,418,182	G	3.24E-07	0.000143	SE
rs134557868	*ACSL6*	7:22,527,204	T	7.16E-06	0.001195	SE
rs385381247	*ACSL6*	7:22,502,848	T	7.16E-06	0.001195	SE
rs29014580	*MRE11*	15:1,472,757	G	7.43E-06	0.001221	SE
rs137215894	*ATP8B4*	10:60,229,145	C	8.72E-06	0.00136	SE
rs132674081	*UBR4*	2:133,508,452	A	1.01E-05	0.001517	SE
rs110095083	*CCDC77*	5:107,313,161	C	1.46E-05	0.001932	SE
rs136092614	*DID O 1*	13:54,507,164	A	1.84E-05	0.002255	SE
rs109892136	*MY O 5C*	10:58,100,890	C	2.26E-05	0.003691	MXE
rs133838482	*NEMP1*	5:56,366,065	C	3.85E-05	0.003762	SE
rs133135962	*DBNL*	22:387,756	T	5.06E-05	0.004496	SE
rs43725274	*ABTB1*	22:59,835,232	A	7.91E-05	0.006175	SE
rs41640891	*ITPR1*	22:21,678,074	C	8.00E-05	0.006228	SE
rs110291055	*NUGGC*	8:10,736,676	C	0.000152	0.008051	A3SS
rs109339682	*PIK3C2B*	16:2,195,385	A	0.000138	0.008948	SE
rs42705933	*PIK3C2B*	16:2,210,365	A	0.000138	0.008948	SE
rs110422856	*NCAPG*	6:37,374,718	G	0.00021	0.011918	SE
rs109978478	*ARAP1*	15:52,256,813	A	0.000439	0.020127	SE
rs41645253	*ZNF397*	24:21,783,988	G	0.000961	0.021919	A5SS
rs110464146	*POGZ*	3:19,350,945	G	0.000552	0.023303	SE
rs133847062	*PRPF8*	19:22,777,382	A	0.000597	0.024564	SE
rs384018186	*ERGIC1*	20:4,689,032	T	0.000762	0.028958	SE
rs43349825	*ERGIC1*	20:4,684,346	G	0.000762	0.028958	SE
rs110293454	*MGAM*	4:105,356,030	A	0.000923	0.032992	SE
rs110477374	*ETV6*	5:98,106,200	G	0.000949	0.033582	SE
rs135381754	*ZSCAN29*	21:55,118,729	A	0.00114	0.038199	SE
rs108957555	*TNRC18*	25:38,925,016	T	0.001053	0.047162	MXE
rs41930956	*ABCA6*	19:61,476,059	G	0.00169	0.049079	SE
rs109376798	*GART*	1:1,997,766	T	0.001692	0.049115	SE

Italicized values in Table 2 represent genes with AS SNPs.

**FIGURE 9:**
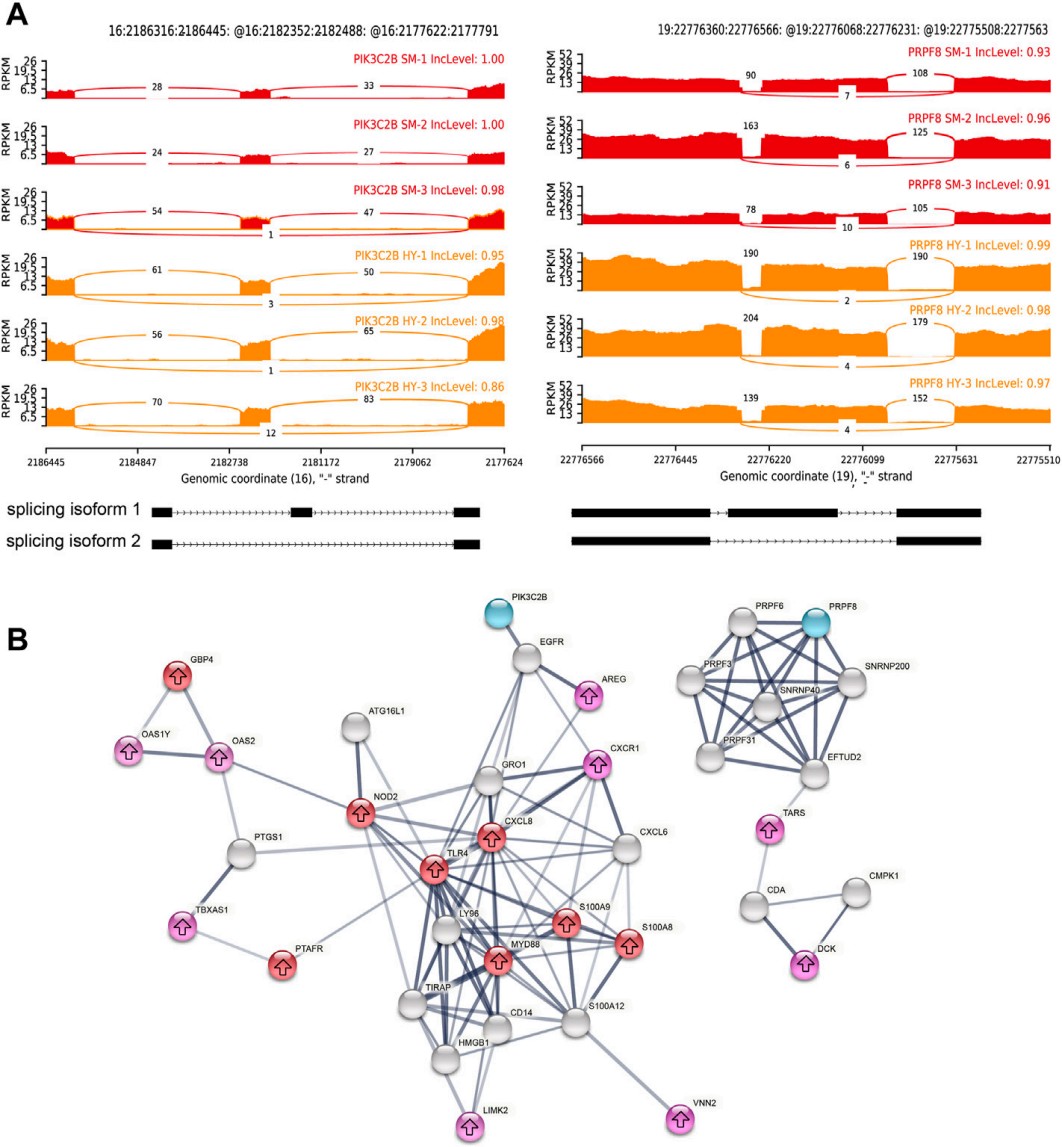
Interacting networks of alternative splicing genes, differentially expressed genes (DEGs), and the target genes of differentially expressed lncRNA (DELs). **(A)** differential alternative splicing (DAS) genes *PIK3C2B* and *PRPF8* exhibited skipping exon (SE) events compared to healthy cows. Red represents healthy cows (HY1, HY2, and HY3) and yellow represents subclinical mastitis cows (SM1, SM2, and SM3). Inclevel represents inclusion level for sample replicates, and gene structure graph represents alternative splicing isoforms imputed from GTF files. **(B)** PPI network of subclinical mastitis resistance-associated genes. Purple nodes represents DEGs, red nodes represents the target genes of lncRNAs that were close to somatic cell count (SCC) and somatic cell score (SCS), and white cell count QTLs, blue nodes represents two DAS genes regulated by SNPs, and gray nodes represents the second shell of interactors. The thickness of the line represents the strength of the interaction, and the arrow represents the up-regulated gene.

To explore the mechanism by which alternative splicing variants regulate subclinical mastitis resistance, it was further investigated the genotype of these 32 AS SNPs between healthy and subclinical mastitis cows. Two AS SNPs were associated with subclinical mastitis resistance, and the genotypes of these AS SNPs were completely dissimilar between the healthy and subclinical mastitis cows (Table 3). Specifically, SNP rs42705933 on chromosome 16 at 2,210,365 bp of gene *PIK3C2B* had the genotype AA in healthy cows and AG in subclinical mastitis cows. Furthermore, SNP rs133847062 on chromosome 19 at 22,777,382bp of gene *PRPF8* had the genotype AC in healthy cows and AA in subclinical mastitis cows (Table 3). Notably, the two DAS genes both presented exon skipping compared to the healthy cows (Figure 9A).

**TABLE 3:** Genotypes of SNP rs42705933 and rs133847062 in two groups.

SNP	REF	ALT	HY1	HY2	HY3	SM1	SM2	SM3
rs42705933	A	G	0/0	0/0	0/0	0/1	0/1	0/1
rs133847062	A	C	0/1	0/1	0/1	0/0	0/0	0/0

Note: 0 represents the reference genomic loci and 1 represents the mutation loci.

To gain more insight into the interactions of these two mastitis resistance-related DAS genes with DEGs, a PPI network was constructed for functional association analysis. As a result, 110 interactions were found to be present in 37 genes (interaction score >0.4). As shown in Figure 9B, some proteins were able to interact, such as *GBP4* could interact with *OAS1Y* and *OAS2*, *MYD88* could interact with *S100A9*, and *TLR4* could interact with *CXCL8*, *NOD2,* and *PTAFR*. On the other hand, one protein interacts with another protein through a third protein. For example, *OAS2* could interact with *TBXAS1* via *PTGS1*, *PIK3C2B* could interact with *AREG* via *EGFR*, and *CXCR1* could interact with *S100A8* via *CXCL6*. The above results suggest that these proteins play a synergistic role in the regulation of bovine subclinical mastitis.

## 4 Discussion

In this study, the expression profiles of mRNAs and lncRNAs from healthy cows and subclinical mastitis cows were characterized, and the functions of 287 DEGs, 70 DELs, and 1,621 DAS genes were annotated, obtaining a number of significantly enriched pathways. Notably, the NOD-like receptor signaling pathway, cytokine-cell receptor interaction, Toll-like receptor signaling pathway, and *S. aureus* infection pathway were also significantly enriched in the available studies of bovine mammary epithelial (BME) cells (Wang et al., 2016; Wang et al., 2020a; Lin et al., 2021), suggesting that these pathways play important regulatory roles in the pathogenesis of mastitis in blood and breast tissue. Subsequently, based on target gene prediction and QTL database annotation, the results showed that the lncRNAs MSTRG25101.2, MSTRG.56327 0.1, and MSTRG.18968.1, which are adjacent to the SCS QTL and SCC QTL, regulate the expression levels of genes *NOD2*, *CXCL8*, OAS2, and *TLR4*, which are involved in the NOD-like receptor signaling pathway, Cytokine-cytokine receptor interaction, and Toll-like receptor signaling pathway.

The innate immune system is the host’s first line of defense against pathogenic microorganisms and relies on pattern recognition receptors (PRRs) (Kawai and Akira, 2010; Celhar et al., 2012; Kawasaki and Kawai, 2014). These PRRs recognize molecular features expressed on microorganisms and the interaction of PRRs with microbe-associated molecular patterns (MAMP) can lead to the expression of pro-inflammatory cytokines and other immunomodulatory molecules (Gilbert et al., 2013). Among several different types of PRRs in cows, Toll-like receptors (TLRs) and NOD-like receptors (NLRs) have been extensively studied (Bhattarai et al., 2018). In the present study, genes *NOD2*, *CXCL8*, *OAS2*, and *TLR4* were differentially expressed in the blood transcriptome of subclinical mastitis cows and involved in the NOD-like receptor, Toll-like receptor pathway and were identified as key candidates for this study. *TLR4* and *NOD2*, as key PRRs, and their recognition of MAMP have been shown to be a key event in the development of mammary inflammation (Goldammer et al., 2004; Aitken et al., 2011; Porcherie et al., 2012). *CXCL8* is an important neutrophil chelator in BME cells (Wellnitz and Bruckmaier, 2012), induces chemotaxis in the target cells, and has a significant change in expression levels upon *TLR4* activation, which could serve as a potential biological for improving the outcome of mastitis markers (Islam et al., 2020).

Ferroptosis is a regulatory form of iron-dependent cell death (Dixon et al., 2012; Zuo et al., 2022), and is characterized by abnormal iron metabolism (Yang and Stockwell, 2016), lethal lipid peroxidation, and reactive oxygen species accumulation. Several studies have demonstrated that ferroptosis plays a vital regulatory role in the pathology of cancers such as lung cancer (Yang and Stockwell, 2016), gastrointestinal cancer (Zhu et al., 2020), and breast cancer (Xu et al., 2021; Zhang et al., 2021). Yet, few studies reported the relationship between ferroptosis and subclinical mastitis of cows, and the precise mechanism of programmed cell death in bovine mastitis is not fully understood. In our study, KEGG results showed that *SLC40A1* (Deng et al., 2021), *SLC11A2* (Weijiao et al., 2021), *ACSL6*, *GCLC* (Chang et al., 2021; Wang et al., 2021), *ACSL5*, *CYBB* (Ping et al., 2022), *VDAC3* (Yang et al., 2020; Zhu et al., 2021)*,* and *FTH1* (Liu et al., 2022) were involved in the ferroptosis signaling pathway (Supplementary Table S4). Among them, *SLC40A1* was up-regulated in subclinical mastitis cows compared to healthy cows (Figure 3B). Moreover, *SLC40A1*, *ACSL6*, *GCLC*, *ACSL5*, *CYBB*, *VDAC3,* and *FTH1* were also DAS genes. Of these, the *SLC40A1* presented SE and RI events and the *FTH1* showed four alternative splicing patterns, including SE and RI, MXE, and A3SS. It is hypothesized that alternative splicing may influence the development of subclinical mastitis by altering the alternative spliced pattern of genes associated with ferroptosis.

RNA splicing is an important regulatory mechanism that links trait-associated variants and complex traits. Alternative splicing generates diverse transcripts with significant roles in disease resistance (Ju et al., 2012a; Asselstine et al., 2022) and the metabolic process (Sun et al., 2021) of the cow. In this study, a total of 2,212 differential splicing events were detected, affecting 1,621 different genes, and GO enrichment analysis showed that they mainly affected some genes that are thought to be involved in transcriptional regulation, proteolytic processes, and neurodevelopment (Figure 8C). It was furthermore reported that the two groups exhibited different AS profiles, with a two-fold increase in the number of SE events in subclinical mastitis cows compared to healthy cows (Figure 7C), whereas there was no significant increase in the number of MXE events, A5SS events and A3SS events, or even a decrease in the number of RI events, demonstrating a link between specific alternative splicing patterns and subclinical mastitis in cows, and similar results were obtained in mammary gland tissues infected with *S. aureus* (Sun et al., 2021). Consequently, we hypothesized that mastitis-specific AS patterns may increase susceptibility to mastitis and that gene-specific AS patterns in the mammary gland of healthy cows may positively influence the disease resistance of cows, which requires further study.

Mutations affected by pre-mRNA splicing account for more than 15% of disease-causing mutations (Caminsky et al., 2014). Previous studies have documented numerous loci of disease susceptibility genes associated with certain specific splicing patterns in cows. For instance, SNPs rs39652267 and rs39631044 in the 3′ flanking region of the gene *JAK2* were dramatically associated with SCC and SCS, and SNP rs43046497 on the intron nine of the gene *STAT5A* was significantly linked to *IL-6* (Usman et al., 2014). These results indicated that the alternative splicing process of mastitis resistance-associated genes is mediated by SNPs. In our study, a total of 32 AS SNPs were identified (Table 2), of which two SNPs rs42705933 and rs133847062 had completely different genotypes between healthy cows and subclinical mastitis cows, which corresponded to SE events of *PIK3C2B* and *PRPF8* (Table 3). Notably, neither of the candidate mastitis genes affected by splice variants in this study, *PIK3C2B* and *PRPF8* were simultaneously regulated at the transcriptional level, indicating independent regulation of genes by alternative splicing, while the PPI interaction network showed that these two alternative splice genes interacted with subclinical mastitis-related genes at the protein level (Figure 9B).

Among them, *PIK3C2B* belongs to class II of the PI3Ks family and performs a pivotal role in the control of membrane trafficking and intracellular signaling (Vanhaesebroeck et al., 2010; Margaria et al., 2019). The splicing factor *PRPF8* is critical for breast cell survival and has potential prognostic value in breast cancer (Cao et al., 2022). What is more, these two SNPs are variants of spliced polypyrimidine fragments (Supplementary Table S5), which are recognized by the polypyrimidine fragment binding protein of the spliceosome complex and are essential for the initial recognition of introns in mammals (Riolo et al., 2021), and the expansion of the polypyrimidine fragment enhances the efficiency of splicing (Romfo and Wise, 1997). Therefore, we hypothesized that the SNPs rs42705933 and rs133847062 may act as pathogenic mutations that cause the genes *PIK3C2B* and *PRPF8* to exhibit exon skipping, which in turn affects the resistance of cows to subclinical mastitis, but the detailed regulatory mechanism remains to be verified in subsequent future studies.

## 5 Conclusion

Our data suggest that genes *TLR4*, *NOD2*, *CXCL8*, and *OAS2* are key components involved in the host immune response to bovine mastitis and that the lncRNAs MSTRG25101.2, MSTRG.56327.1, and MSTRG.18968.1 adjacent to the SCC QTL and SCS QTL may potentially regulate expression of these candidate genes. In addition, characterization of peripheral white blood cell transcriptome alternative splicing events indicated that specific types of alternative splicing events appear to be associated with mastitis resistance in cows and that single nucleotide polymorphisms in the DAS genes *PIK3C2B* and *PPRF8* may be risk factors for mastitis susceptibility in cows. The genes and lncRNAs highlighted in this study could serve as expression biomarkers for mastitis that can be used for genetic improvement of dairy cattle for resilience to mastitis. In conclusion, our study provides a basis for further investigation of the molecular mechanisms of resistance and susceptibility to subclinical mastitis in cows.

## Data Availability

The datasets presented in this study can be found in online repositories. The names of the repository/repositories and accession number(s) can be found below: https://www.ncbi.nlm.nih.gov/, PRJNA839517.
